# Cell type-specific expression of Eps8 in the mouse hippocampus

**DOI:** 10.1186/1471-2202-15-26

**Published:** 2014-02-17

**Authors:** Chiung-Chun Huang, Yun-Shen Lin, Cheng-Che Lee, Kuei-Sen Hsu

**Affiliations:** 1 Department of Pharmacology, College of Medicine, National Cheng Kung University, Tainan 701, Taiwan

**Keywords:** Eps8, Calbindin, Cholecystokinin, Interneuron, Hippocampus

## Abstract

**Background:**

Epidermal growth factor receptor substrate 8 (Eps8) is a multifunctional protein that regulates actin cytoskeleton dynamics and architecture through its barbed-end capping and bundling activities. In cultured hippocampal neurons, Eps8 is enriched at dendritic spine heads and is required for spine morphogenesis; however, the detailed expression pattern of Eps8 in the hippocampus has not yet been explored.

**Results:**

Here, we demonstrate that endogenous Eps8 protein is restrictively expressed in neurons (NeuN-positive), but not in glial cells (glial fibrillary acidic protein-positive) in area CA1 of the mouse hippocampus. Surprisingly, Eps8 immunoreactivity is rarely found in pyramidal cell somata, but is expressed predominantly in the somata and dendrites of 67 kDa isoform of glutamic acid decarboxylase-expressing GABAergic interneurons in the stratum radiatum and at the border of stratum radiatum and lacunosum-moleculare of area CA1. On the basis of co-localizing markers, we found that Eps8 is not present in perisomatic inhibitory parvalbumin-expressing cells or calretinin-expressing interneurons. However, Eps8 is richly expressed in calbindin-expressing interneurons. Furthermore, Eps8 is also present in cholecystokinin-expressing interneurons, but not in somatostatin-expressing interneurons in area CA1 stratum pyramidale and stratum radiatum.

**Conclusions:**

These results reveal a previously unknown cell type-specific expression pattern of endogenous Eps8 protein in the mouse hippocampus and speculate that the role of Eps8 in controlling and orchestrating neuronal morphogenesis and structural plasticity might be more prominent in interneurons than in pyramidal cells of the hippocampus.

## Background

Epidermal growth factor (EGF) receptor substrate 8 (Eps8), a member of the Eps8-family proteins, is originally identified as a substrate for the EGF receptor tyrosine kinase [1]. It contains a putative N-terminal phosphotyrosine binding protein (PTB) domain, a central Src homology 3 (SH3) domain and a C-terminal effector domain, each being a potential site for protein-protein interaction [2]. Previous structural and functional studies have revealed that Eps8, via its SH3 domain, participates in forming distinct protein complexes that either transduce signals from Ras to Rac leading to actin remodeling or regulate endocytosis of receptor tyrosine kinases [2-4]. In addition, it may regulate actin dynamics through its ability to activate Rac by forming an active complex with the guanine nucleotide exchange factor Sos-1, the adaptor Abi-1, and the p85 regulatory subunit of phosphoinositide 3-kinase [5], the stability of which is increased by association with insulin receptor substrate p53 [6]. Furthermore, Eps8 also directly controls actin cytoskeleton dynamics and architecture via its actin barbed-end capping and actin bundling activities, which resides in its C-terminal effector domain [5]. While the molecular characteristics of Eps8 have been elucidated over the past few years, most functional studies of this protein were performed in fibroblasts or cancer cell lines. The significance of Eps8 in neurons; however, has only just begun to be elucidated.

Regarding the expression of Eps8 in the brain, it has been shown that Eps8 is specifically localized to the somatodendritic and axonal compartments of granule cells and unipolar brush cells in the rat cerebellum [7]. Furthermore, Eps8 is expressed in both synaptosomal and postsynaptic density fractions and is tightly associated with postsynaptic density (PSD) proteins, PSD-95, chapsyn 110/PSD-93 and *N*-methyl-D-aspartate (NMDA) receptor subunit NR1, in cerebellar granule cells [7,8]. Interestingly, recent studies show that Eps8 contributes to the formation of dendritic spines and activity-mediated synaptic plasticity in cultured hippocampal neuron model [9] and Eps8-null mice exhibits a defect in spine formation and learning-dependent spinogenesis in the hippocampus [10]. Furthermore, Eps8 has also been shown to regulate axonal filopodia formation in cultured hippocampal neurons in response to brain-derived neurotrophic factor [11]. These results strongly suggest an important role for Eps8 in the regulation of hippocampal neuronal structure and function. However, there is scarce information about the endogenous expression pattern and cellular distribution of Eps8 in the hippocampus. Here, we analyze the expression pattern of endogenous Eps8 protein in the mouse hippocampus using fluorescence immunohistochemistry and confocal microscopy. Our data suggest that Eps8 protein in the hippocampus is predominantly expressed in calbindin- and cholecystokinin-expressing GABAergic interneurons in area CA1 stratum radiatum and the border of lacunosum-moleculare.

## Results

### Immunolocalization of endogenous Eps8 in area CA1 of the mouse hippocampus

**Figure 1:**
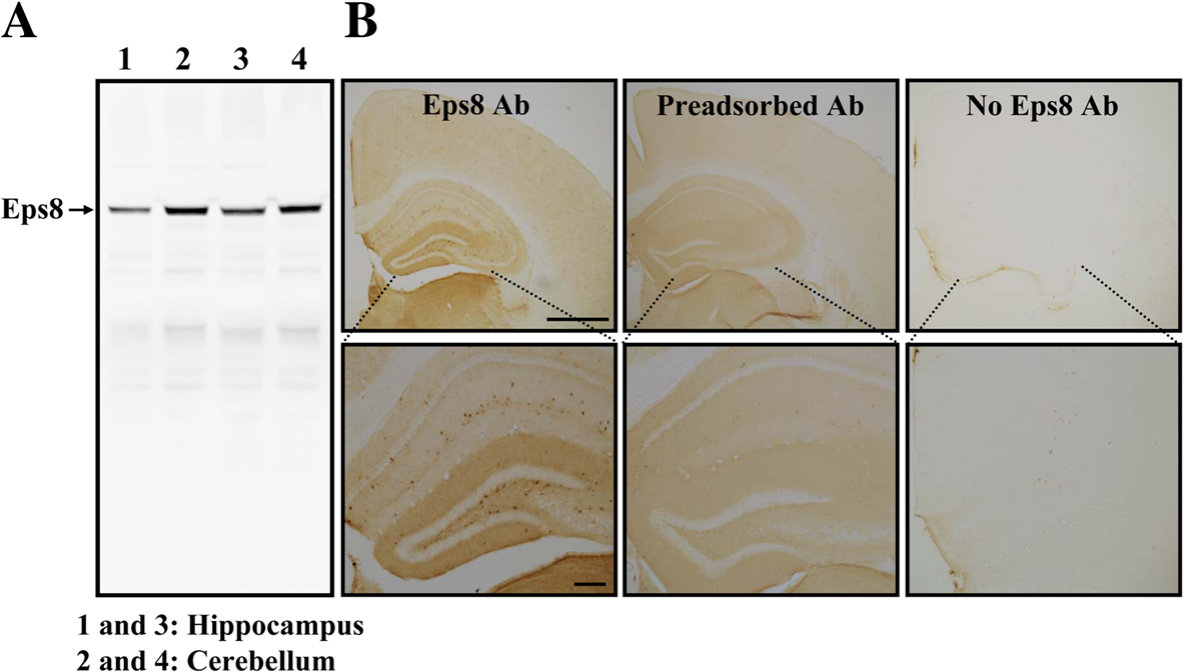
**Specificity of anti-Eps8 antibody confirmed by Western blotting and immunohistochemistry. (A)** Representative immunoblot showing the expression of Eps8 protein in the mouse hippocampus and cerebellum. The applied antibody recognized a major band corresponding to the Eps8 protein mass of ~97 kDa. **(B)** Representative coronal sections of the mouse brain stained with primary anti-Eps8 antibody (left), preadsorbed antibody (middle), or without (right) primary antibody (top panel). The hippocampal region is shown at higher magnification on the bottom panel. Similar results were obtained in 4 mice. Scale bars, 1 mm for upper panel and 200 μm for bottom panel.

To verify the specificity of the Eps8 antibody, Western blot analysis of hippocampal and cerebellar tissue lysates was conducted. The Eps8 antibody specifically detected a band at ~97 kDa, consistent with the molecular weight of Eps8 protein (Figure 1A). To identify the Eps8-expressing cells in the hippocampus, we first performed immunohistochemical staining with anti-Eps8 antibody in hippocampal sections. To our surprise, Eps8 immunoreactivity was rarely found in pyramidal cell layer lying in area CA1, but appeared moderate in the cell bodies and dendrites of cells located in the stratum radiatum and at the border of stratum radiatum and lacunosum-moleculare (Figure 1B, left). We also performed control experiments where the primary antibody was preadsorbed with the blocking peptide (Figure 1B, middle) or omitted (Figure 1B, right). Control experiments showed no immunostaining, further confirming the specificity of the Eps8 antibody.

**Figure 2:**
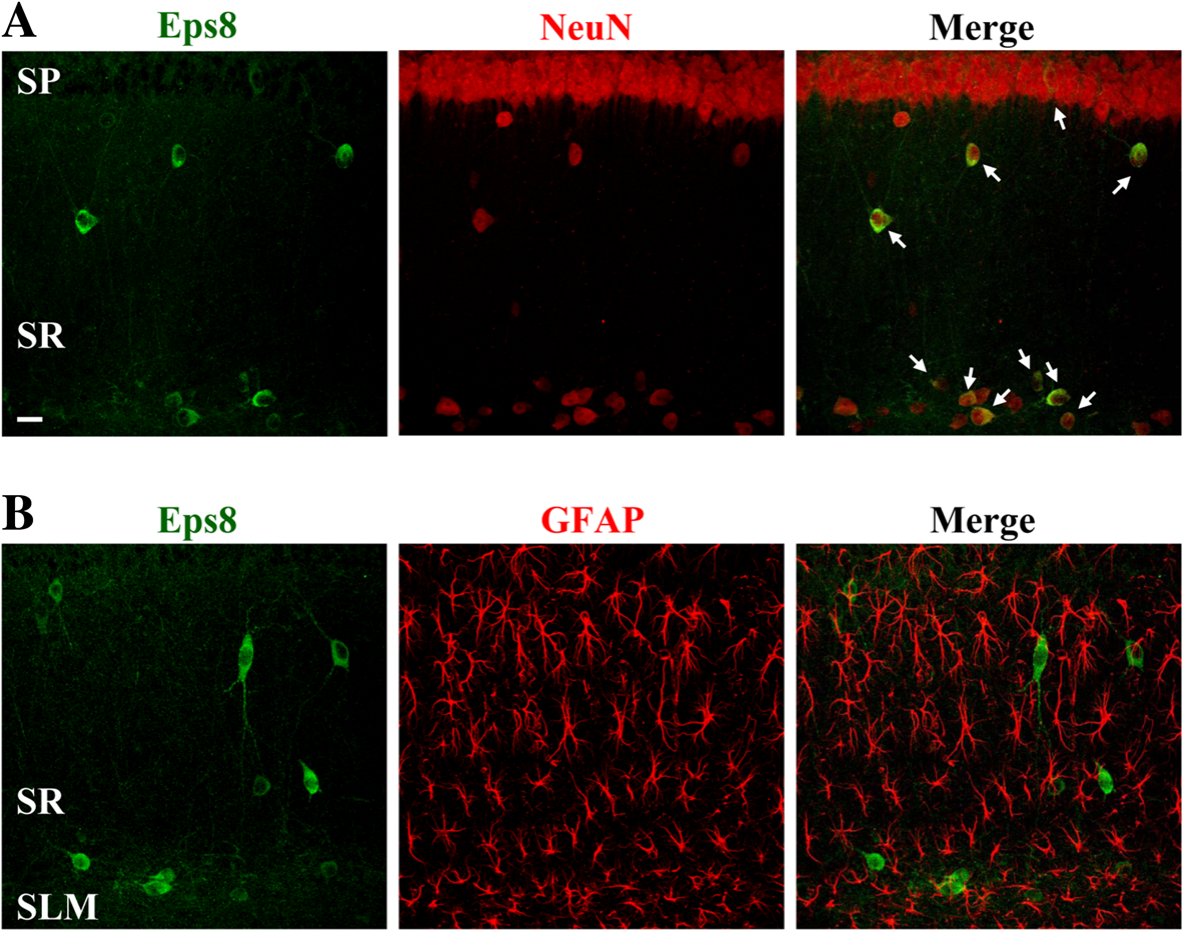
**Eps8 is specifically expressed in neurons. (A)** Doubled-labeled confocal immunofluorescent images showing the colocalization of Eps8 (green) and the neuronal marker NeuN (red) in area CA1 of the mouse hippocampus. The merged image indicates that all Eps8-immunopositive cells express NeuN. Arrows point to double-labeled cells. **(B)** Doubled-labeled confocal immunofluorescent images showing the lack of any colocalization between Eps8 (green) and the glial marker GFAP (red). Similar results were obtained in 4 mice. Sacle bar, 20 μm.

Double immunofluorescent staining with the mature neuronal marker NeuN revealed that nearly all of the Eps8-expressing cells were positive for neuronal nuclear antigen (NeuN; Figure 2A). In contrast, we did not detect Eps8 immunoreactivity in glial cells, which were identified by antibody directed against the glial marker glial fibrillary acidic protein (GFAP) and showed typical star-shaped astrocyte morphology (Figure 2B), indicating that Eps8 expression is essentially confined to neurons.

### Specific subpopulations of hippocampal interneurons expressing Eps8

**Figure 3:**
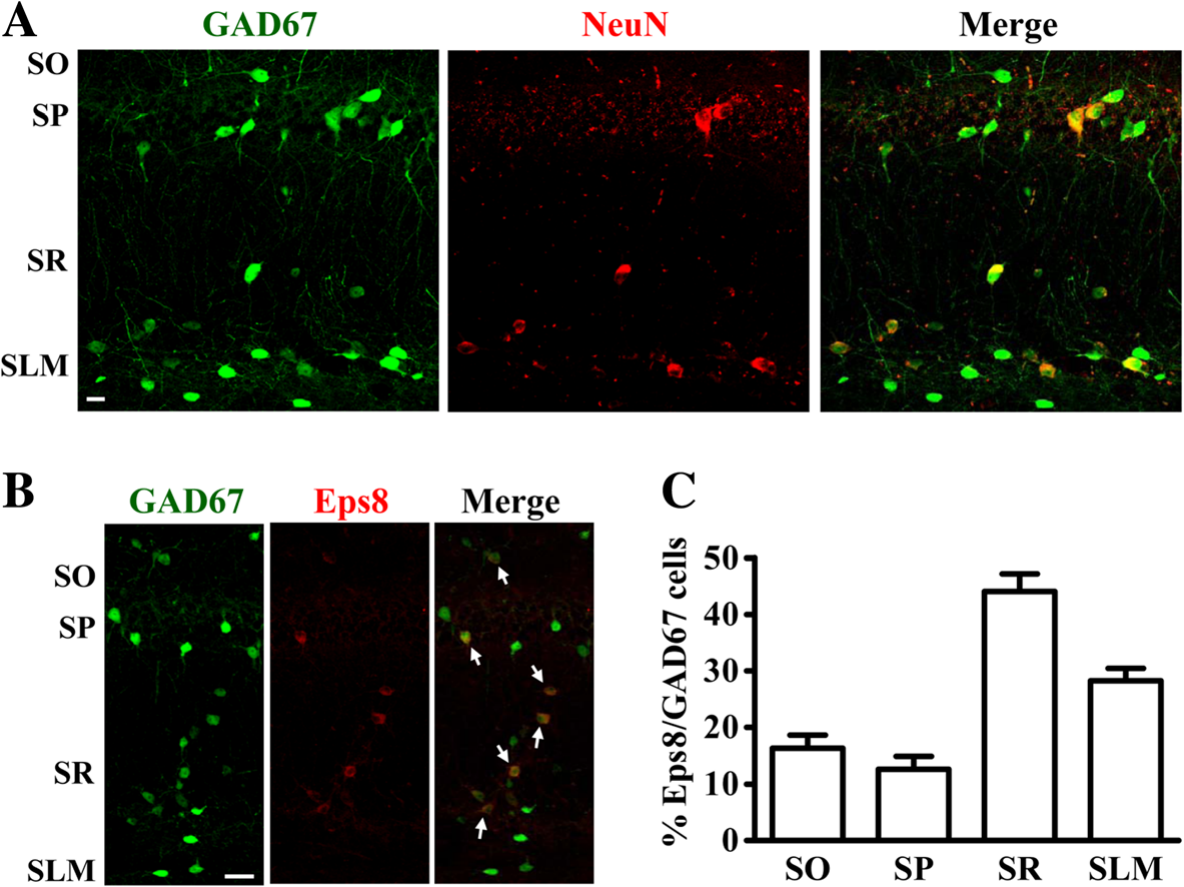
**Eps8 is specifically expressed in GAD67-expressing cells. (A)** Doubled-labeled confocal immunofluorescent images showing the colocalization of GAD67 (green) and NeuN (red) in hippocampal area CA1 of GAD67-GFP knock-in mice. **(B)** Doubled-labeled confocal immunofluorescent images showing the colocalization of GAD67 (green) and Eps8 (red) in hippocampal area CA1 of GAD67-GFP knock-in mice. The merged image indicates that all Eps8-immunopositive cells express GAD67. Arrows point to double-labeled cells. **(C)** Bar graph showing the percentage of GFP-labeled interneurons that expressed Eps8 in hippocampal area CA1 of GAD67-GFP knock-in mice. Similar results were obtained in 4 mice. Scale bar, 20 μm.

Given that glial cells do not express Eps8, the morphology and localization of the intense Eps8 immunoreactivity in the stratum radiatum and the border of lacunosum-moleculare imply that those Eps8-expressing cells are most likely interneurons. We therefore set out to characterize Eps8-expressing cells in hippocampal area CA1 of 67 kDa isoform of glutamic acid decarboxylase-green fluorescence protein (GAD67-GFP) knock-in mice, in which GABAergic interneurons are specifically labeled with GFP fluorescence. In agreement with the previous report [12], GFP-expressing interneurons were present in all layers of hippocampal area CA1 and displayed immunoreactivity for GABA (data not shown) or NeuN (Figure 3A), confirming their identity as GABAergic interneurons. As expected, the majority of Eps8-expressing cells were GFP-expressing interneurons (Figure 3B). The proportion of GFP-expressing interneurons coexpressing Eps8 was highest in the stratum radiatum (44%) and less in the stratum lacunosum-moleculare, stratum oriens and stratum pyramidale (28%, 16% and 13%, respectively; Figure 3C).

**Figure 4:**
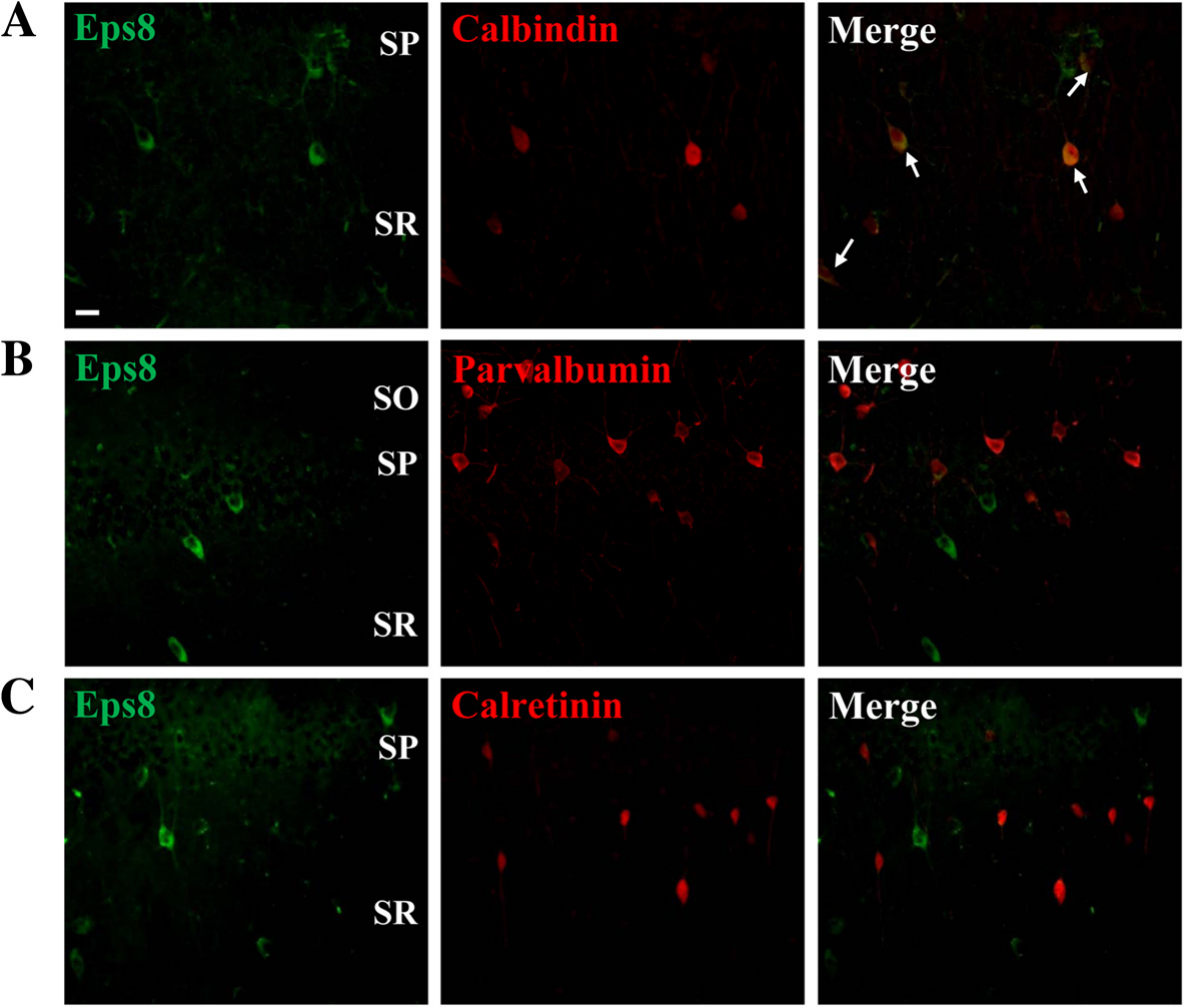
**Eps8 is specifically expressed in calbindin-expressing interneurons. (A)** Confocal immunofluorescent image of calbindin-expressing interneurons located in the stratum pyramidale (SP) and stratum radiatum (SR) (left). Confocal immunofluorescent image of immunolabeling for Eps8 in the same section (middle) and the merged image (right) indicates that calbidin-expressing interneurons express Eps8 protein (arrow). **(B)** Doubled-labeled confocal immunofluorescent images showing that parvalbumin-expressing interneurons in the stratum oriens (SO), SP and SR do not express Eps8 protein. **(C)** Doubled-labeled confocal immunofluorescent images showing that parvalbumin-expressing interneurons in the SP and SR do not express Eps8 protein. Similar results were obtained in 4 mice. Scale bar, 20 μm.

To further assess the Eps8-expressing interneuron subpopulation, co-staining of Eps8 with subpopulation-specific interneuronal markers was conducted. We performed immunofluorescent staining for calbindin, parvalbumin and calretinin in hippocampal sections. Double-labeling for Eps8 and calbindin revealed that all Eps8-expressing cells were positive for calbindin (Figure 4A). However, no Eps8-expressing cells were found that expressed parvalbumin (Figure 4B) or calretinin (Figure 4C).

**Figure 5:**
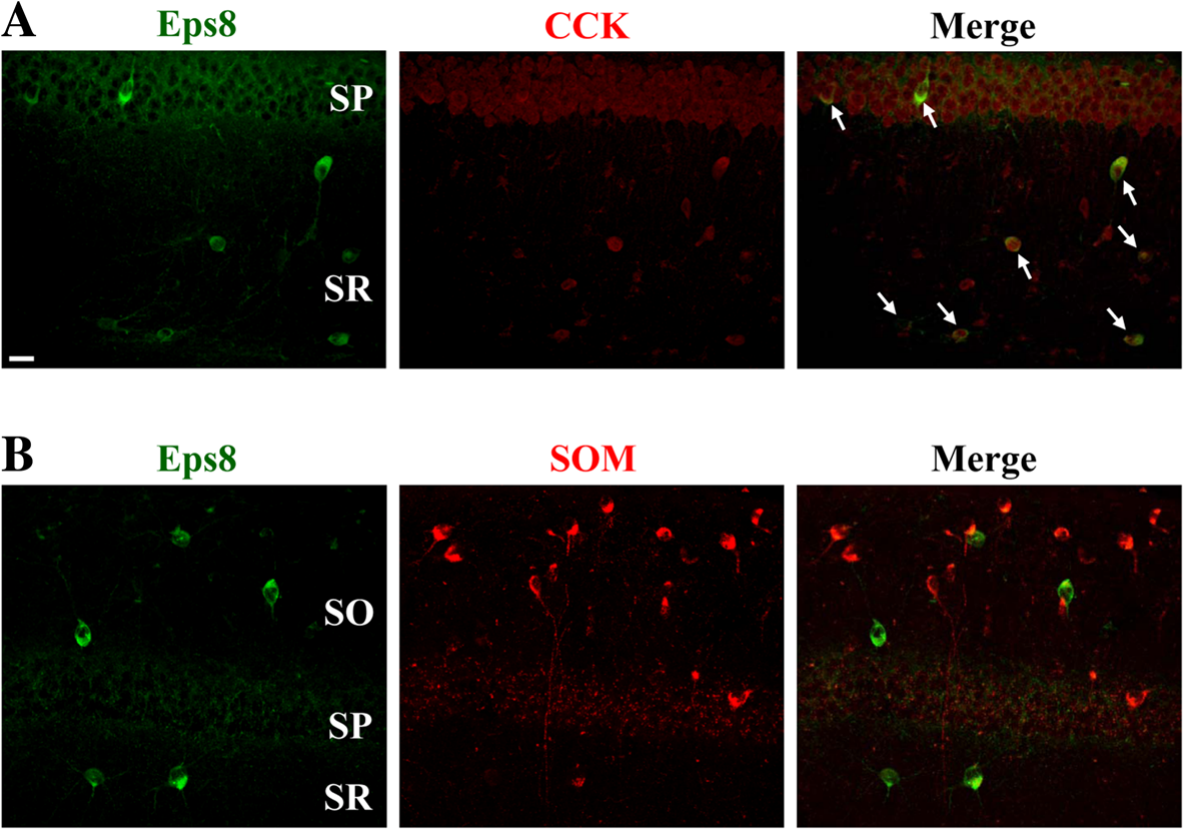
**Eps8 is specifically expressed in cholecystokinin (CCK)-expressing interneurons. (A)** Confocal immunofluorescent image of CCK-expressing interneurons located in the stratum pyramidale (SP) and stratum radiatum (SR) (left). Confocal immunofluorescent image of immunolabeling for Eps8 in the same section (middle) and the merged image (right) indicates that CCK-expressing interneurons express Eps8 protein (arrow). **(B)** Doubled-labeled confocal immunofluorescent images showing that somatostatin (SOM)-expressing interneurons in the stratum oriens (SO), SP and SR do not express Eps8 protein. Similar results were obtained in 4 mice. Scale bar, 20 μm.

We next examined in more detail the molecular profile of Eps8-expressing cells in hippocampal area CA1. The vast majority of Eps8-expressing cells were positive for cholecystokinin (Figure 5A). We did not observe colocalization of Eps8 protein with somatostatin (Figure 5B).

Finally, interneurons immunopositive for each maker were quantified according to their soma location within hippocampal CA1 layers and then the propotion of interneurons in each layer, which colocalized with Eps8 was assessed (Table 1). The proportion of calbindin-expressing interneurons coexpressing Eps8 was in highest in the stratum radiatum (88%), less in the stratum lacunosum-moleculare and stratum oriens (64% and 4%, respectively), and null in stratum pyramidale. Likewise, the majority of cholecystokinin-expressing interneurons coexpressing Eps8 were located in the stratum radiatum (55%) and stratum lacunosum-moleculare (53%), and only 4% of these interneurons located in the stratum oriens.

**Table 1:** **Coexpression of Ca**^**2+**^**-binding proteins and peptides with Eps8 protein in CA1 interneurons of the mouse hippocampus**

	**Number of cell in layer/total**				**% coexpression with Eps8**			
	**SO**	**SP**	**SR**	**SLM**	**SO**	**SP**	**SR**	**SLM**
Parvalbumin	35/67	30/67	2/67	0/67	0	0	0	0
Calretinin	10/57	18/57	20/57	9/67	0	0	0	0
Calbindin	25/72	1/72	24/72	22/72	4	0	88	64
Cholecystokinin	82/238	2/238	66/238	88/238	13	0	55	53
Somatostatin	72/78	5/78	1/78	0/78	3	0	0	0

SO, stratum oriens; SP, stratum pyramidale; SR, stratum radiatum; SLM, stratum lacunosum-moleculare.

## Discussion

There is emerging evidence that Eps8 is involved in the regulation of activity-mediated spine formation, possibly through its actin barbed-end capping and bundling activities [9,10]. Mice lacking Eps8 display abnormal growth of immature spines and cognitive impairment [10]. Our findings provide, to the best of our knowledge, the first demonstration for a cell type-specific expression pattern of endogenous Eps8 protein in the mouse hippocampus. Our data indicate that in area CA1 Eps8 protein is predominantly expressed in calbindin- and cholecystokinin-expressing GABAergic interneurons in the stratum radiatum and the border of lacunosum-moleculare. We further show that Eps8 expression is rare in CA1 pyramidal cell somata, and glial cells do not express Eps8.

Literature regarding the role of Eps8 in the formation of dendritic spines and axonal filopodia comes mainly from the hippocampal neuronal culture system in overexpression condition [9-11]. It has been shown that Eps8 expressed in both the cell bodies and neurites, and prominently enriched in the dendritic spine heads and axonal growth cones of cultured hippocampal pyramidal neurons [9,11]. With the same anti-Eps8 antibody as was used in the current study, a previous study has demonstrated the expression of Eps8 protein in scattered cells of CA1, CA3 and dentate gyrus of the mouse hippocampus [8]; however, they did not identify which types of cells express Eps8 protein. In this study, we have extended these findings by showing that Eps8 is restrictedly present in some subpopulations of hippocampal neurons, but not in glial cells. It is worth noting that only few Eps8-expressing cell bodies were found in CA1 stratum pyramidale of the mouse hippocampus. Most Eps8-expressing cells located in the stratum pyramidale also contain GAD67, indicating that they belong to GABAergic interneuron classes. Furthermore, it is unlikely that the weakness of Eps8 immunoreactivity observed in CA1 pyramidal cells results from insufficient detection sensitivity of Eps8 antibody used in the current study. This view is supported by (i) under our staining condition, we successfully detected abundant expression of Eps8 protein in specific CA1 interneuron subpopulations, and (ii) strong immunoreactivity to Eps8 antibody was detected in mouse cerebellar neurons (data not shown), which are known to constitutively express high levels of Eps8 [7]. These observations would then be logical to assume that endogenous regulation of neuronal morphogenesis and structural plasticity by Eps8 in the hippocampus might be more significant in interneurons than in pyramidal cells.

With respect to the subcellular localization, Eps8 protein has been reported to be expressed both presynaptically in the molecular layer and postsynaptically in the glomeruli of the granule cell layer in adult mouse cerebellum [7,8]. Although we did not explore this issue in detail in this study, our immunolocalization data indicate that Eps8 protein is expressed in both soma and dendrites of a subset of GABAergic interneurons in area CA1 of the mouse hippocampus. Further studies are required to examine its subcellular localization patterns with specific biomarker staining for subcellular compartments.

Using GAD67-GFP knock-in mice and immunofluorescent double-labeling, we confirmed that some subpopulations of GABAergic interneurons in hippocampal area CA1 express high levels of Eps8. Eps8-expressing interneurons were present in all layers of area CA1 with the higher density in the stratum radiatum and at the border of stratum radiatum and lacunosum-moleculare. With respect to the calcium-binding proteins and peptides that characterize different subpopulations of GABAergic interneurons [13-15], we found substantial co-expression of Eps8 and calbindin, but not parvalbumin or calretinin. We also found that Eps8 is present in a subset of cholecystokinin-expressing interneurons. Taken these findings into consideration, our results suggest that Eps8-expressing cells in hippocampal area CA1 predominantly belong to the calbindin- and cholecystokinin-expressing subpopulations of GABAergic interneurons. In addition to using molecular expression profiles, GABAergic interneurons can be distinguished on the basis of their morphology, electrophysiological features, and innervations of distinct subcellular domains of pyramidal cells [16,17]. So far, more than 20 different types of interneurons have been recognized in the hippocampus and neocortex [18]. It has been reported previously that CA1 pyramidal cells are supported by at least 16 distinct types of GABAergic interneurons, each having highly stereotypic laminar arrangements and functions [19,20]. Considering the pattern of co-staining and distribution, we speculate that Eps8-expressing cells belong to a s of Schaffer collateral-associated interneurons, which preferentially innervate pyramidal cell apical dendrites in conjunction with the Schaffer collateral-commissural input pathways [21]. Functionally, Schaffer collateral-associated interneurons have a regular accommodating firing pattern and provide a feedforward inhibition onto CA1 pyramidal cells [21,22]. These findings raise the intriguing possibility that Eps8-expressing GABAergic interneurons may act in concert with other classes of GABAergic interneurons to innervate distinct domains of pyramidal cells, thereby controlling and orchestrating hippocampal network activities.

What could be the function of Eps8 expression in GABAergic interneurons? Given its ability to regulate the organization of the actin cytoskeleton [5], it seems reasonable to speculate that Eps8 may play some role in maintaining GABAergic interneuron morphogenesis. Another possible role for Eps8 is in coordinating dynamic processes of membrane receptor trafficking [2]. Indeed, a mechanistic role for Eps8 in mediating clathrin-mediated endocytosis of EGF receptors and fibroblast growth factor receptors has been reported in HeLa cells [3,4]. Since constitutive and regulated trafficking of membrane receptors to and from the plasma membrane are important processes for the maintenance of neuronal function and responsiveness to external stimuli, the specific expression of Eps8 in GABAergic interneurons may be critical for maintaining the homeostatic levels of membrane receptors. Additional cell type-specific control of Cre-mediated Eps8 conditional knockout studies are needed to examine these possibilities.

## Conclusion

Our study identifies novel expression of Eps8 protein in specific interneuron subpopulations in the mouse hippocampus, in addition to its known expression in the dendritic spine heads and axonal growth cones of glutamatergic pyramidal cells. Further work is needed for the detailed molecular and electrophysiological analyses to measure the functional significance of newly characterized Eps8-expressing cells in the hippocampus.

## Methods

### Animals

Male 10–12 weeks old wild type C57BL/6 and GAD67-GFP knock-in mice [12] were used in our experiments. GAD67-GFP knock-in mice were generously provided by Dr. Yuchio Yanagawa and bred within our animal facility onto the C57BL/6 genetic background. Mice were group housed in a humidity- and temperature-controlled (25 ± 1°C) vivarium on a 12 h light/dark cycle with access to food and water ad libitum. All experiments were executed in accordance with the National Institutes of Health Guide for the Care and Use of Laboratory Animals and were approved by the Institutional Animal Care and Use Committee of National Cheng Kung University.

### Immunohistochemistry

Mice were deeply anesthetized with sodium pentobarbital (50 mg/kg, intraperitoneally) and perfused transcardially with phosphate-buffered saline (PBS) and 4% paraformaldehyde. After the perfusion, brains were removed and continue to fix in 4% paraformaldehyde for 24 h at 4°C and then transferred to the solution containing 30% sucrose that immersed in 4°C for at least 48 h before slicing. Coronal brain slices containing the hippocampus were sectioned to a 20 μm thickness, washed with 0.3% Triton X-100, and then incubated for blocking with solution containing 3% goat serum in PBS. The sections were incubated in the primary antibodies: mouse anti-Eps8 (1:1000; BD Biosciences, San José, CA); mouse anti-NeuN (1:1000; Chemicon, Temecula, CA); rabbit anti-GFAP (1:2000; Dako, Carpinteria, CA); rabbit anti-parvalbumin (1:1000; Millipore, Billerica, MA); rabbit anti-calbindin (1:500; Millipore); rabbit calretinin (1:500; Millipore); rabbit anti-somatostatin (1:500; Millipore); rabbit anti-cholecystokinin (1:500; Millipore). Finally, sections were washed with 0.3% Tween 20 in PBS and then incubated with the secondary Alexa Fluor 488 or Alexa Fluor 568 antibodies (Molecular Probes, Eugene, OR) for 2 h at room temperature. For the preadsorption control, anti-Eps8 antibody was mixed with a 5-fold excess (wt/wt) of blocking peptide (containing the epitope recognized by the antibody, catalog # sc-4236, Santa Cruz Biotechnologies, Santa Cruz, CA) for 30 min at room temperature before being used for immunolabeling. In some experiments, biotinylated horse anti-mouse immunoglobulin (1:1000; Vector Elite ABC kit, Vector Laboratories, Burlingame, CA) and streptavidin-horseradish peroxidase complex were applied, followed by 3,3′-diaminobenzidine (Sigma-Aldrich, St. Louis, MO) until a brown reaction product was observed. The immunostained sections were collected on separate gelatin-subbed glass slides, rinsed extensively in PBS, and mounted with ProLong Gold Antifade Reagent (Invitrogen, Carlsbad, CA). Fluorescence images of neurons were obtained using an Olympus FluoView FV1000 confocal microscope with sequential acquisition setting at a resolution of 1024 × 1024 pixels and a sampling of six consecutive optical sections in the Z-stack. The high magnification images were recorded with an Olympus Plan Apochromat 60× oil-immersion objective (1.42 numerical aperture and 0.15 working distance). All images were imported into NIH ImageJ software (National Institutes of Health, Bethesda, MD) for analysis, and all the parameters used were kept consistent during capturing. To verify the specificity of antibodies, control experiments were done by omitting primary antibody and, in each case, no visible staining was detected (Figure 1B).

Interneurons immunopositive for each marker within hippocampal CA1 region were quantified in images from about 1.5 to 2.5 mm posterior to Bregma every sixth coronal section by using the optical fractionator sampling method [23,24]. A counting frame (300 × 600 μm square) was sampled per CA1 region in each section. Neurons were quantified by counting 6 sections per mouse and were counted only when the soma was in clear immunostaining within the counting frame. The boundaries of the hippocampal regions were manually delineated in accordance with the description by Franklin and Paxinos [25].

### Western blotting

The hippocampus and the cerebellum were dissected and homogenized in ice-cold Tris-HCl lysis buffer (TBS; pH 7.4) containing a cocktail of protein phosphatase and proteinase inhibitors as described previously [26]. Samples were sonicated and spun down at 15,000 × g at 4°C for 10 min. The supernatant was then assayed for total protein concentration using Bio-Rad Bradford Protein Assay Kit (Hercules, CA). Each sample from tissue homogenate was separated in 8% SDS-PAGE gel. Following the transfer on nitrocellulose or polyvinylidene fluoride membranes, blots were blocked in buffer solution containing 5% milk and 0.1% Tween-20 in PBS (in mM: 124 NaCl, 4 KCl, 10 Na_2_HPO_4_ and 10 KH_2_PO_4_; pH 7.2) for 1 h and then blotted for 2 h at room temperature with anti-Eps8 antibody (1:1000; BD Biosciences). It was then probed with horseradish peroxidase-conjugated secondary antibody for 1 h and developed using the ECL immunoblotting detection system (Amersham Biosciences, Buckinghamshire, UK), according to manufacturer’s instructions.

## Abbreviations

EGF: Epidermal growth factor; Eps8: Epidermal growth factor receptor substrate 8; GABA: γ-Aminobutyric acid; GAD67-GFP: 67 kDa isoform of glutamic acid decarboxylase-green fluorescence protein; GFAP: Glial fibrillary acidic protein; NeuN: Neuronal nuclear antigen; NMDA: *N*-methyl-D-aspartate; PSD: Postsynaptic density; PTB: Phosphotyrosine binding protein; SH3: Src homology 3; TBS: Tris-HCl lysis buffer.

## Competing interests

The authors declare no competing financial interests.

## Authors’ contributions

CCH, YSL and CCL performed the experiments and the statistical analysis. CCH, YSL and KSH designed the study and wrote the manuscript. All authors read and approved the final manuscript.
